# Bluey, Requestival, Play School and ME@Home: the ABC (Kids) of communication cultures during lockdown

**DOI:** 10.1177/1329878X20952520

**Published:** 2021-02

**Authors:** Liz Giuffre

**Affiliations:** University of Technology Sydney, Australia

**Keywords:** ABC online, broadcast, children, communication, culture, lockdown, radio, television, Triple J, young people

## Abstract

When a nationwide lockdown was declared in Australia in March 2020, the role of the ABC as the public broadcaster became vital. Unprecedented pressure was placed on parents and carers as families were cut off from their physical networks and communities beyond immediate household groups. This article focuses on the specialist material created and curated by the ABC to entertain, educate and continue to provide cultural connection for households with children and young adults, particularly broadcast and post-broadcast outlets ABC Kids, ABC ME and Triple J. Notably, these outlets were able to provide both a connection to the ‘real world’ and ‘real events’ happening outside during this time, but they were also able to provide materials to escape and appease audience anxiety pitched at a level that is age appropriate.

## Introduction

When a global pandemic was declared in March 2020, the ABC was where many members of the Australian public again turned for information, advice and comfort. Using the youth outlets Triple J, ABC ME and ABC Kids, the ABC created and curated materials that supported the specific needs of young people during the period of unprecedented physical isolation. This included the provision of news pitched to age appropriate levels of comprehension, as well as entertainment and other cultural materials. These outlets ensured that young Australians were able to feel connected despite being physically unable to gather together in their usual groups at school, day care, work or play.

This article is organised around three central themes – education, entertainment and culture. The outlets considered are ABC’s dedicated children’s and young people’s networks ABC Kids (for pre-schoolers), ABC ME (school-aged children) and Triple J (teenagers and young adults). Existing education models, such as Harrison’s (2004) excellent ‘Entertainment, Engagement and Empowerment’ method of exploring educational children’s television, serve as further points of departure to examine the longer lasting effects of materials made and consumed during COVID-19’s initial fallout. However, I am mindful of the importance of considering these programmes in terms of the entertainment and cultural connections they provided for their audiences, particularly during times of extreme stress as has been the case during the COVID lockdowns. Using ‘culture’ to encapsulate ‘a way of life’ (Williams in Barker, 2002: 66) is vital when considering how young and very young audiences have coped with this period of physical isolation from their normal peer and extended family networks. Returning to Stuart Hall’s (1990) definition of the role of cultural and media studies to ‘enable people to understand what is going on, and especially provide ways of thinking, strategies for survival, and resources for resistance’ (p. 22), here I consider the way the ABC provided outlets for young people to ‘do’ things with each other. I argue this has been a vital service provided by the national broadcaster during this very difficult time.

When discussing the role of broadcast and post-broadcast media it is important to remember that there is a clear digital divide in Australia. Children and young people living in rural areas, outside major capital cities, in households with low incomes, in households with specialist accessibility needs and in places with large Indigenous and migrant communities have all been shown to be at a significant disadvantage in terms of affordable access to digital resources (Thomas et al., 2020: 6). In the early days and weeks of the COVID-19 pandemic, charitable organisations like the Father Bob Maquire Foundation responded to the needs of children now left stranded in digital poverty. Similar to the ADII’s findings, Father Bob’s study found that digital poverty was “especially prevalent among disadvantaged groups of children from Indigenous backgrounds, CALD communities, low income families and for people living in public housing”’ (Fatherbobs.com, 2020: online) resulting in a sharp immediate barrier for these young people to access schooling while in lockdown. The ABC’s continued presence across traditional free to air broadcast outlets, as well as through free and accessible online post-broadcast and digital first platforms, provided essential equity services for students with varied digital access circumstances across the country. Without the national broadcaster’s provision of a range of services across a range of platforms, huge numbers of young Australians would be completely cut off beyond their immediate households. The urgent need for connection during the COVID-19 lockdown period from March to June 2020 was unpredented in many ways, but the role of the national broadcaster in providing diverse and accessible crisis support can be compared to the services provided by the ABC during natural disasters like floods and fires (Freeman et al., 2018)

## Education – COVID-19 purpose built

One of the most discussed parts of life during the March to June 2020 lockdown period was the experience of ‘home schooling’ and/or ‘distance learning’.^1^ When schools physically closed students across the country (and it seemed the world) were left to rely on materials delivered to them online. This process placed a huge burden on everyone involved, as qualified classroom teachers had to quickly adapt and often create new materials to be delivered solely online, while also teaching their students (and parents/carers) how to learn in this way. The results were obviously widely varied according to the resources that each individual had at their disposal, and the experience brought into sharp focus the digital divide between Australian citizens and regions (for early results, see Varadharajan, 2020).

ABC outlets responded to school closures with the provision of formal education materials to supplement the broadcaster’s existing suite of services and further support the delivery of school curricula. In late-April 2020, the Victorian Minister for Education, James Merlino (2020), declared a formal partnership with the broadcaster, stating his support for ‘[p]artnering with ABC to provide learning materials via TV will give students a new way to learn and help keep them engaged as they learn from home’ (online), to be recognised through the state’s ‘Learning From Home’ and its associated ‘FUSE’ (Find, Use, Share, Education) websites.^2^ Individual materials on the ABC site were also linked to the NSW Department of Education^3^ and reference to selected ABC Kids materials was also made on the South Australian Department of Education site.^4^

ABC outlets had been developing and providing educational materials prior to the COVID-19 lockdown. Housed at the online portal www.abc.net.au/education, these included links to television and radio broadcasts, apps, podcasts, online first and only resources and curated social media. The ABC online outlet is part of the broadcaster’s long history of providing educational support (Giuffre, 2013; Griffen-Foley, 2019, 2020; Harrison, 2011). What was different this time was the extensive and express link made between programming and formal teaching, notably with the ‘Teaching Resources’ provided on the ABC TV Education website (2020b: online). As of 18 June 2020, there were 40 programmes and associated resources listed on this site, many including resources for multiple episodes (ABC TV Education, 2020b). The programmes included early childhood and lower primary targeted pieces such as *Play School Storytime* (a spin-off of the traditional ‘Play School’ franchise featuring Australian celebrities reading picture books); upper primary and early high school for long-running programmes like *BTN* (*Behind the News*) and the BTN themed specials called *BTN Media Literacy*; and upper high school targeted programmes such as *Fall in Love With Music, Poetry Between the Lines* and *The House in Session* (each aligned with HSC equivalents). Also available as part of this list were items adaptable across a broad formal teaching spectrum, such as the notes relating to the ABC ME programme *Social Media Me*, which the teacher’s notes relate directly to the NSW PDHPE K–10 Syllabus which can encompass Kindergarten all the way through to year 10 (just pre-HSC) (ABC TV Education, 2020a: online).

**Figure 1.**
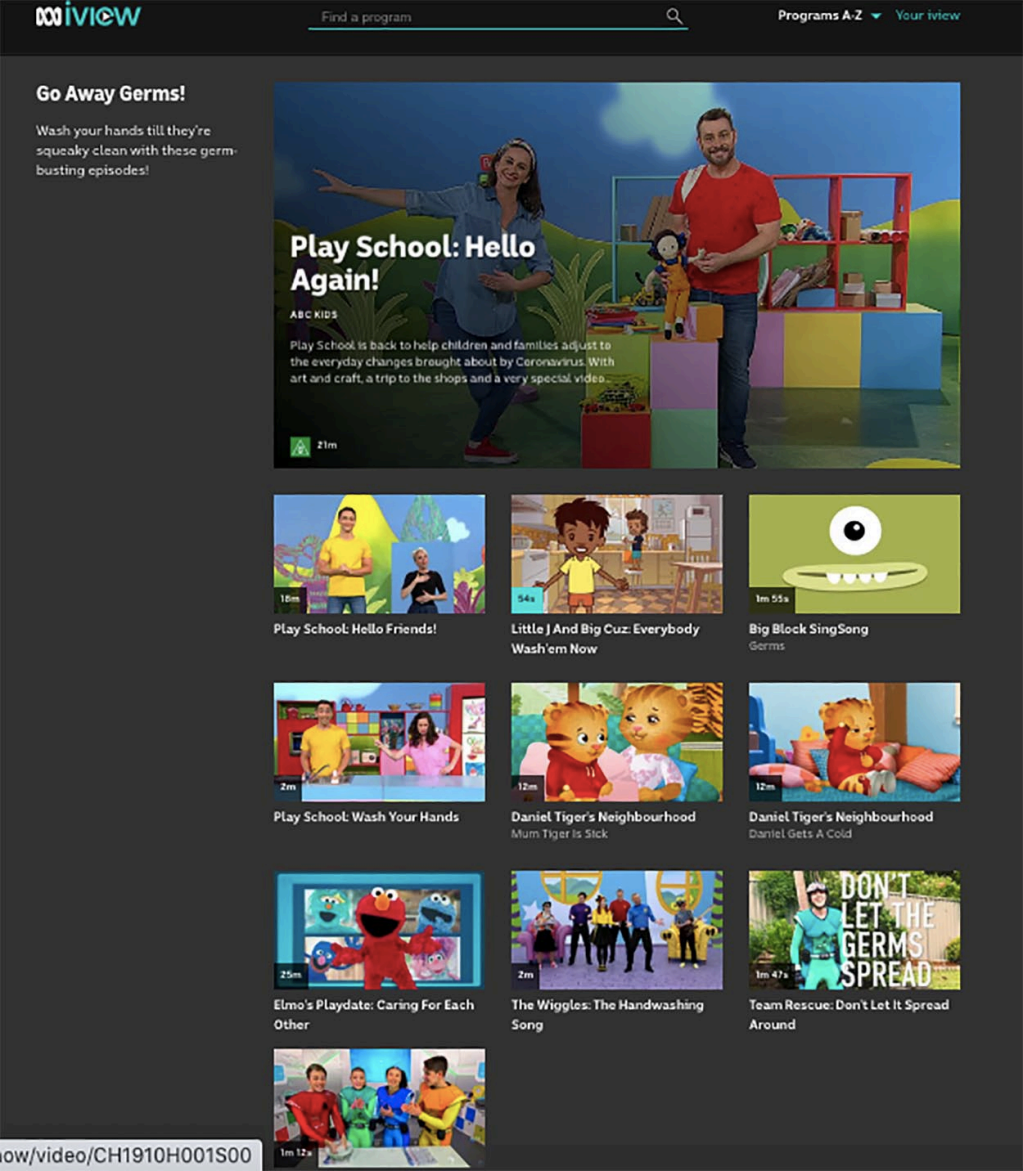
Screenshot taken from ABC iview showing programmes curated under the ‘Go Away Germs!’ title, 18 June 2020, https://iview.abc.net.au/collection/2429.

In addition to these formal links between school curriculum and programming, items were organised and added to the ABC platforms in a way to help young people and carers identify them as educational. For example, on the ABC Kids section of the ABC iview portal, videos relating specifically to the public education campaigns for COVID-19 have been organised as part of a ‘Go Away Germs’ subcategory (see Figure 1). These videos all appeared on the digital platform either at the same time, or shortly after, their original broadcast on the station’s broadcast channel ABC Kids.

The ‘Go Away Germs’ collection included specials from ABC commissioned programmes such as *Play School* and The Wiggles, as well as co-productions like *Little J and Big Cuz* (with NITV/SBS) and international specials like the *Sesame Street* offshoot *Elmo*’*s Playdate: Caring for Each Other* (PBS/HBO America). Of the 11 videos featured in this section of iview, half directly related to public health education, featuring different songs and musical rhymes directing young viewers to wash their hands. Three videos were specially created items made as part of the Play School franchise, including a ‘Wash Your Hands’ music video and new song of that name, and two special 21-minute episodes of the programme called ‘Hello Friends!’ and ‘Hello Again!’, designed specifically to address young Australians who were newly experiencing the lockdown period. Described on the ABC iview (2020b) platform as a special ‘to help children and families adjust to the everyday changes brought about by the Coronavirus’ (online), both specials and additional content relating to Play School were compiled to support the broadcast and online features. The special ‘Notes for Families and Educators’ publication featured talking points to support further discussion, sheet music to the original song ‘Wash Your Hands’ for families to play themselves at home, suggestions for games to play relating to public health and mental health concerns, and practical ways to continue to connect with family and friends during lockdown like ‘creating email journals for grandparents’ (Stone, 2020: 4). Importantly, the document’s authors were careful to detail the educational underpinning of the children’s programme and its messaging, stating that the *Play School* Specials had been ‘Created as co-viewing opportunities (in careful collaboration with early education advisors and the ABC Science Unit)’ so as to ‘assist parents and educators to explain COVID-19 to children and support young families adjust to the unprecedented challenges the global community is facing’ (Stone, 2020: 1).

*Play School* is one of the few Australian children’s shows to receive sustained academic attention during its time on air, with studies acknowledging the show’s commitment to representing diversity in Australia (Mackinlay and Barney, 2008), promoting engagement through play (Van Vliet et al., 2013) and even as a way to track the changing media environment in Australia more broadly over its many decades on air (Harrison, 2012). *Play School* again proved to be an innovative outlet for Australian children’s entertainment and education during the first months of COVID-19, as specials like ‘Hello Friends!’ and ‘Hello Again!’ provided continuity for young Australians whose other routines may have been swiftly changed when lockdown measures came into effect. The use of regular presenters during these specials, as well as regular Play School characters like Big Ted, Little Ted, and a single piano soundtrack to underpin the dialogue and songs, provided a familiar way to address young viewers and their families. Even though the very contemporary messaging in the programmes themselves were very unusual for the show (*Play School* rarely makes mention of current events), this combination of usual format and extraordinary circumstances allowed for a way to communicate a public education message with young viewers and their families in a highly accessible and appropriate way for the specific needs of these Australians. Importantly, the mixture of traditional broadcast and post-broadcast online catch-up delivery ensured the largest possible number of Australians could access this content.

## Entertainment – COVID-19 repurposed (and sometimes purposely ignored)

**Figure 2.**
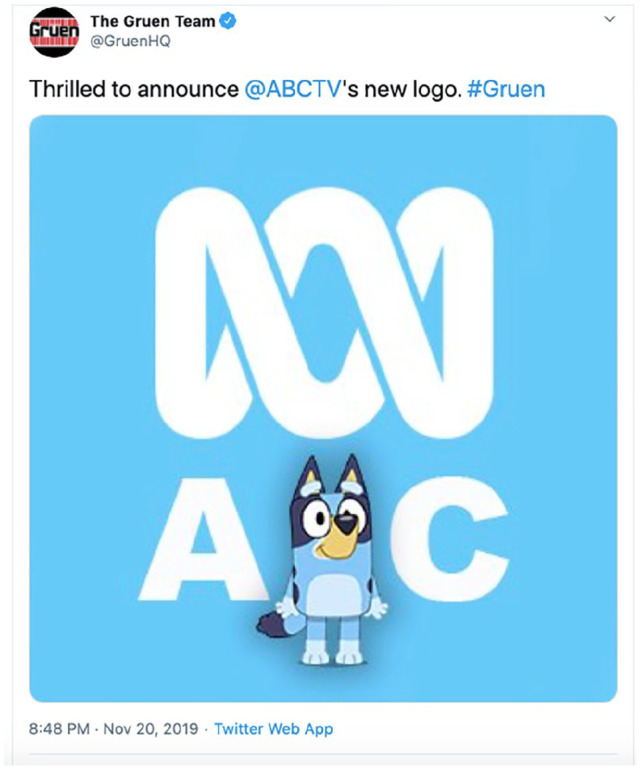
Screenshot of tweet from ‘Gruen HQ’, https://twitter.com/GruenHQ/status/1197089346689490944 (accessed 18 June 2020).

The ABC issued a media release for 17 March entitled ‘Breaking Bluey News’ (ABC Media Release, 2020: online). Referring to the launch of the second series of the Australian-made award-winning pre-school animation by Brisbane’s Ludo Studio (in conjunction with BBC Worldwide, Screen Australia and Screen Queensland and the ABC), the *Bluey* media release was significant because its date also marked the first official day of lockdown for many Australian households. The show, based on the lives of a family of Blue Heeler dogs, had caught the attention of the mainstream media beyond its intended ABC Kids demographic. In terms of content, *Bluey*’*s* success can be attributed to its distinctively Australian landscapes and cultural markers as much as its more ‘traditional’ children’s television markers like strong stories and characters. *Bluey*’*s* success prior to the lockdown had already been well established with accolades from industry and audiences, including its status as the most successful iview item ‘of all time’ at the end of its first season in 2018/2019 (ABC Media Release, 2020: online) and with the achivement of an International Emmy win. Wil Anderson, comedian and host of long-running ABC TV programme Gruen, suggested only partly in jest in November 2019 that the children’s show had been so successful that the ‘B’ in ‘ABC’ actually stood for ‘Bluey’ (Knox, 2019: online) – see also Figure 2.

It appears to have been a co-incidence that the second season of *Bluey* was released on the day lockdown was declared by Prime Minister Scott Morrison. *Bluey*’*s* creators, Ludo Studios, did engage with the COVID-19 crisis by releasing a special ‘wash your hands’ version of one of the show’s original songs, ‘Poor Little Bug on the Wall’ (Goulis, 2020as: online); one of many children’s shows that released these types of paratexts to communicate public health messages to young audiences education tools similar to the ‘go away germs’ collection on ABC Kids cited above. Early media reports have also suggested that *Bluey* provided relief for families in lockdown beyond Australia too. For example, *The New York Times* ran a feature on the show in April 2020, leading with the headline focused on lockdown watching – ‘Stuck Inside? Here’s an Australian Kids’ Show Every Parent Can Love’ (Sebag-Montefiore, 2020: online).

**Figure 3.**
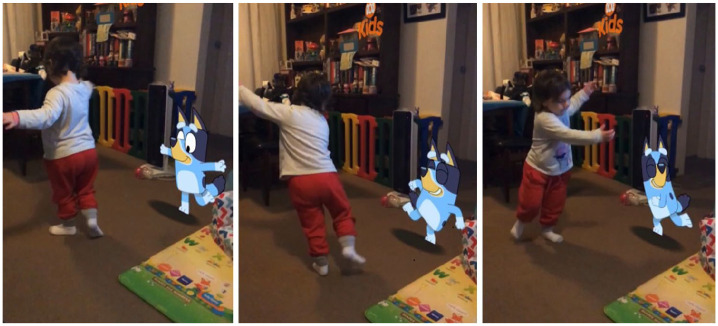
‘Dance with Bluey’ screenshots created by author on Facebook, 28 May 2020.

Although a co-incidence rather than a pre-planned campaign, the *Bluey* episodes released in the March 17 group provided a satisfying escape from what was happening in the locked down country (or, as the characters in the show would say, what was happening ‘for real life’). This escape was perhaps most effective with episodes where *Bluey*’s characters engaged in lockdown prohibited activities. For example, Episode 1, ‘Dance Mode’ saw the family eating out at a café followed by an outdoor festival; episode 2, ‘Hammerbarn’ featured a long and very non-socially distanced trip to the local hardware store; episode 3, ‘Featherwand’ was a game inspired by Bluey leaving to go to a birthday party; episode 4, ‘Squash’ featured team sport and episode 5, ‘Hairdressers’ a pretend outing to the hairdresser. As the series continued, other episodes also featured activities pre-school viewers could relate to but not directly undertake during COVID-19 restrictions, notably episode 8 ‘Daddy Dropoff’ (about going to day care) and episode 11 ‘Cherades’ (where Bluey and her sister, along with their cousins, visit their Grandmother). In addition to watching the show itself young viewers were also encouraged to continue to participate with the show’s characters and activities as extended forms of play, with entertainment, rather than education or awareness, a primary concern. For example, Ludo and the ABC released the ‘Dance with Bluey’ Facebook filter, modelled on the episode ‘Dance Mode’. The filter encouraged viewers and their families to take videos of themselves dancing with the show’s protagonist and upload these to the site for play on the station (see Figure 3, ABC Kids, 2020: online). Another activity was the official *Bluey* website’s simple instructions showing viewers and families how to draw or create home-made versions of favourite episode themes or games.^5^

In an interview with *Bluey* executive producer Daley Pearson, broadcaster Carrie Bickmore described the second season as ‘godsend’ for families ‘a lifeline during [the] pandemic’ (Bickmore in Fowler, 2020: online), and parenting website calling the show ‘The therapy we all need’ and an essential way of ‘getting families through covid lockdown’ (Patience, 2020: online). These testimonies are just a couple of examples demonstrating the show’s value in providing an entertaining escape for its viewers rather than a reminder of the 2020 events. As commentators, journalists and mother/fans of the show Mary Bolling and Kate McMahon explained on the show’s official podcast, *Gotta Be Done*, these references to activities that were now out of bounds for families were enjoyable, but also bittersweet. During a discussion shortly after the airing of ‘Cherades’, the commentators praised the episode’s narrative, comedy and characterisation while also noting the disconnection between the idealised world of the show and the COVID-19 reality – ‘cue nationwide tears as we all miss our grans [grandparents]’ (Bolling and McMahon, 2020: online).

## Culture and community – COVID-19 redeveloped, defined and combined

The ABC’s broadcast and post-broadcast services also extended to older children and young adults during the initial lockdown period. Of particular note is the specially made broadcast/online/app programme *ME@Home* by ABC ME, and Triple J’s week long radio/online/app event ‘Requestival’. These outlets also featured public health announcements and entertainment through techniques similar to those mentioned above, but in this section, I will focus on how these initiatives served to maintain a sense of community and cultural connection for their audiences. For teenagers and young adults, the fostering of culture and community was particularly vital, as these were groups whose senior schooling and early career employment had been either greatly changed, stalled or in the extreme halted altogether. This group of young people were also identified as the most vulnerable to mental health damage during times of stress, with research demonstrating the importance of maintaining peer and other community networks in order to help maintain wellness in this area.

The 10-minute ‘magazine-style’ programme *ME@Home* began on 20 April 2020 and featured simple ways for a school-aged audience to engage with each other and their shared experiences while housebound. Like *Play School* and *Bluey*, the show was regularly broadcast on ABC TV (at an approximate end-of-school time of 3 pm), and also made available for catch-up on demand and online. The anticipated success of the show as an innovative piece of television saw its launch reported as far away as British music magazine NME, who described the *ME@Home* as featuring ‘iso gameshow, celebrity guests and boredom busters from young people across the country’ (Lim, 2020: online). NME’s interest was also due to the Australian musicians featured in the show, with international award winners Tones and I, Samantha Jade and G Flip all used in the show’s reporting and imagery (Lim, 2020). In this way, a connection to youth, music and a regular magazine-style community can be drawn back all the way in ABC TV’s history to shows like the pre-*Countdown* music show *GTK* (*Get to Know*), which was also made for young people as daily 10-minute episodes (Giuffre, 2009: 35−37).

*ME@Home* provided a significant cultural and community connection for otherwise isolated Australian school-aged children. The show’s hosts and guests all referenced being at home and the challenges this posed in terms of staying motivated and well, often discussing ways to deal with these issues while spending time in isolation from their peers. Described on ABC iview (2020a) as ‘a daily fast-paced whip around the country to see how kids and celebs are staying entertained in isolation’ (online), main host Grace Koh opened episode *ME@Home* talking direct to camera from various locations around her house. There was clearly lower quality vision and sound than would normally be featured in a studio-based television show, however, each episode was still slickly edited, featuring fast-paced cut aways to pop culture memes (often American icons like *The Simpsons* and Hollywood teen films), multiple guests and brightly coloured on screen text and labels. The resulting combination of apparently do-it-yourself (DIY) content with tight post-production allowed young viewers to feel their experiences in isolation were being represented. The show’s content also gave audiences something to aspire to beyond their currently (mundane) experience in lockdown. Special episodes at the end of each week encouraged viewers to participate with a regular quiz as well as send in segments to be played on the show. The resulting audience-generated content came of an open invitation on the *ME@Home* website where viewers were asked to send videos in with the pitch ‘How are you busting your boredom while you’re spending so much more time at home? Got any great tips, tricks, hacks or snacks you’re loving at the moment? We want to see!’ (ABC Me, 2020: online). *ME@Home* had produced 40 episodes as of 20 June 2020, with another show also commissioned by ABC ME to also address this audience and coping during lockdown, a mock news programme aimed at young Australians called *Definitely Not News*.

## Entertainment and a culture of musical connection – Triple J’s Requestival

Triple J’s ‘Requestival’ was held over a week from 25 to 31 May 2020 (B&T Magazine, 2020). Another specialist outlet developed by the ABC in response to the COVID-19 restrictions, the concept was to hand the station over to its listeners exclusively for their requests for that period. There were some exceptions (the official ‘Requestival’ was only held from 6 am to 9 pm each day), but for the most part the event allowed Triple J listeners on broadcast, online or on the station’s app to directly dictate what music was played by the station. In a piece on the Triple J website a week before Requestival launched, music producer Newstead wrote a history of request programmes on radio and their ability to bring audiences, professionals and amateurs together. Declaring request shows (and events) are ‘not just a transactional relationship . . . [ they are] about the interaction’ (Newstead, 2020d: online), he continued by explaining ‘[t]o request makes us human – it separates us from the animals (and algorithms’ (Newstead, 2020d). The last point about creating a type of experience invited young audiences to actively participate with each other rather than just with a medium. As opposed to the other types of ‘connection’ that young people may find – curated playlists or recommendations generated on social media or streaming services, participants for Requestival were prompted to make individualised connections to music when they lodged their requests to the station. These connections were confirmed as requesters were required to nominate an artist and song title for their request, followed by a line to simply explain ‘why’ they had made this choice. There were literally thousands of experiences shared on air through the week-long event, with a notable example of these connections being requests and reasons connected to a song by Geelong band ‘Louie The Milk Man’. Although the song itself was not particularly remarkable, and it had never been played on the station before, a group of the band’s friends and fans requested the song be played as a tribute to the band’s singer who had suicided only shortly before the Requestival event. Triple J played the song on its morning programme on the second day of Requestival and also presented a newstory on its website further connecting the song to the story behind its request. In addition to these the station also promoted links to further assistance for any listeners who may be in need of mental health support (Newstead, 2020b: online).

During Requestival Triple J reported receiving ‘105,430 requests . . . over 26,831 new downloads of the triple j app’ and ‘38,000 texts across the week’ (Newstead, 2020a: online). B&T Magazine followed up on the event by calling it a ‘proven a success for the broadcaster, with engagement going through the roof’, noting ‘almost 71 per cent of requestors so far have been under 30, with almost half (48 per cent) aged under 24’ (B&T 2020: online). While this was only a fraction of number of people estimated to participate in station’s annual Hottest 100 poll,^6^ the variety of music requested during Requestival revealed a variety of types of connections made by the young listenership and a more nuanced engagement than the more famous event. While the Hottest 100 has been criticised for merely reflecting already dominant styles of music and identity (Strong, 2010), Requestival featured a wide variety of music including a range of genres, eras and artists, as well as novelties like ringtones and television themes (Earp, 2020: online; Newstead, 2020a; Radioinfo, 2020: online).

The challenge to have music not usually played on Triple J was part of the Requestival appeal for many participants and listeners. The most requested song for Requestival was ‘Duel of the Fates’ by John Williams from the 1999 Star Wars film *The Phantom Menace* (Rose, 2020: online). The station has not published the reasons listeners gave for making this request, however, the song was likely chosen because of the escapism and nostalgia it evoked, as well as its connection to existing online communities as a much-circulated video and cover on YouTube (Greiving, 2017: online). ‘Duel of Fates’ was also something of a joke among the existing community of Triple J listeners, having been requested repeatedly for the ‘Bump day’ segment on the Veronica and Lewis Drive programme for the station. Finally, as part of presenter Veronica’s last day on air, the song was played, with listener comments also replayed and recalled on Triple J online (Bracken, 2020: online). Although the Requestival event, and the COVID-19 lockdowns, are both unprecedented, Triple J listeners have previously connected with each other through songs, often as ‘insider jokes’. One famous example of such a connection was when Pauline Pantsdown’s satirical ‘I’m a Backdoor Man’ reached the 1997 Hottest 100 after only a few days play on air (Johnson, 2003).

Requestival was held at a time when young Australian adults were particularly socially (and economically) isolated. With the financial impact of lockdown measures likely to hit this group most immediately and with the least structural support, there was a real need to create a generational culture of solidarity, if nothing else. Although the station did not explicitly say it was using the event to reconnect listeners with their peers and something bigger than their immediate circumstances, the enthusiasm of responses on and beyond that station’s broadcast, post-broadcast and associated outlets showed these connections were clearly made. For example, Reddit users discussed the festival in real time with each other and shared memories and experiences sparked by the song’s played on air, with Reddit user ‘Tranquilbez22’ posting full daily lists of each Requestival. Tranquilbez22 (2020) labelled their list on Requestival Day 7 as ‘[a]n absolute honour making these lists and listening to the radio all week. Fantastic distraction from the global chaos’ (online), with comments underneath his thread on that day, and the six-preceding, full of praise for the effort made and chance to follow Requestival with such depth. The online version of Australian Rolling Stone also featured a daily ‘highlights’ report of the first 5 days of the event (Jenke, 2020: online), however, peer-to-peer outlets like the Reddit thread (with its public comments section) provided more scope for connections between audience members.

**Figure 4.**
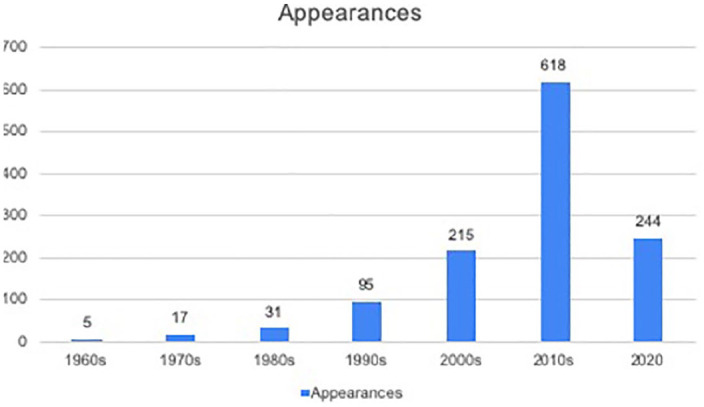
Broadcast requests from Requestival by decade.

I have conducted a preliminary audit of the 1225 songs played as part of the week long Requestival, gathering data as published on Triple J’s ‘Recently Played’ webpage (https://www.abc.net.au/triplej/featured-music/recently-played/). Selecting each day and timeslot relating to Requestival event (from 25 May to 31 May 2020, between 6 am and 9 pm), I have then manually compiled data relating to key aspects of each song, such as release date, artist’s country of origin, and genre. While there is much more to be done here, and this analysis only represents the songs that were selected for broadcast, as opposed to the reportedly 70,000 requests that were received for the festival (Newstead, 2020c), there are already interesting patterns to be observed. For example, there was a large concentration of music from the 2010s played, as well as very recent music from 2020 (see Figure 4). This trend suggests that audiences were wanting to connect with peer groups and recent memories of music, perhaps music they had seen live or experienced first hand as new releases rather than through relatives or archives. There were also significant number of requests played featuring female artists – at least 360, or just over 29%. When compared with the percentage in other Triple J ‘events’ like the historic Hottest 100 (Strong, 2010), this shows an upwards trend towards gender equity of representation for the station.

## Conclusion

All Australians were able to turn to the ABC to educate, inform and entertain them during the period of intense lockdown between mid-March and early-June 2020. The services provided to young and very young Australians through ABC multiplatform outlets during lockdown were particularly innovative, diverse and engaging, serving to help ease the effects of the economic, social and cultural losses that had been rapidly delivered. The programmes featured in this article also demonstrate the responsiveness of a national broadcaster that itself was working during a period of financial crisis. The variety of targeted opportunities provided, catering for many access levels, was impressive.

While programmes with already established support like *Play School* and *Bluey* should continue to thrive, it will be interesting to see how ‘one offs’ like *ME@Home* and Requestival are considered and perhaps replicated again. In the post-COVID period, I hope that those young viewers from this time will continue to engage (and engage fondly), with the touchstones the broadcaster provided during this time. Future research will also reveal what lasting influences these programmes had, not just to ‘keep the wheels turning’ at the time, but in the way these young people grow to consider communication cultures in times to come.

## Notes

1. I acknowledge that there is a difference between these two concepts, with ‘home schooling’ technically referring to a pre-existing formal method of approved learning, while ‘distance learning’ has come to be the preferred way that delivery of school materials during COVID-19 emergency conditions has been described.

2. At the time of writing these were being updated regularly. For more information, see https://fuse.education.vic.gov.au/ResourcePackage/ByPin?pin=8YFJ8Z and https://www.education.vic.gov.au/about/department/Pages/learningfromhome.aspx.

3. For example, the teaching resource relating to the episode of *Play School Storytime* featuring Julia Zemiro reading ‘Where The Forest Meets The Sea’ features NSW Department of Education branding and references to its lower primary syllabus (ABC TV Education, 2020c) (https://www.abc.net.au/cm/lb/12276330/data/play-school-story-time-forest-data.pdf, accessed 18 June 2020).

4. https://www.education.sa.gov.au/our-learning-sa/prior-school/abc-story-time-little-library.

5. These were uploaded regularly at www.bluey.tv, but also circulated through the ABC Kids social media pages and those for Ludo Studio, as well as other partner broadcasters and fans. The official Bluey TV website remains the best reference for them, particularly the pages https://www.bluey.tv/make/ and https://www.bluey.tv/play/ (accessed 18 June 2020).

6. Although numbers for this vary each year, the ABC Annual Report for 2019 stated ‘2.7 million votes cast for the Hottest 100 that year, with “An estimated 3.2 million people engaged with the Hottest 100 countdown on 27 January”’ (p. 30).
